# The Alphavirus Exit Pathway: What We Know and What We Wish We Knew

**DOI:** 10.3390/v10020089

**Published:** 2018-02-22

**Authors:** Rebecca S. Brown, Judy J. Wan, Margaret Kielian

**Affiliations:** Department of Cell Biology, Albert Einstein College of Medicine, Bronx, NY 10461, USA; rebecca.brown@einstein.yu.edu (R.S.B.); judy.wan@phd.einstein.yu.edu (J.J.W.)

**Keywords:** alphavirus, assembly, budding, cell-to-cell transmission, intercellular extensions

## Abstract

Alphaviruses are enveloped positive sense RNA viruses and include serious human pathogens, such as the encephalitic alphaviruses and Chikungunya virus. Alphaviruses are transmitted to humans primarily by mosquito vectors and include species that are classified as emerging pathogens. Alphaviruses assemble highly organized, spherical particles that bud from the plasma membrane. In this review, we discuss what is known about the alphavirus exit pathway during a cellular infection. We describe the viral protein interactions that are critical for virus assembly/budding and the host factors that are involved, and we highlight the recent discovery of cell-to-cell transmission of alphavirus particles via intercellular extensions. Lastly, we discuss outstanding questions in the alphavirus exit pathway that may provide important avenues for future research.

## 1. Introduction

The alphavirus genus belongs to the *Togaviridae* family and contains ~30 virus species [1,2]. Medically relevant alphaviruses include Venezuelan, Western, and Eastern Equine Encephalitis viruses (VEEV, WEEV, and EEEV), Ross River virus (RRV), and Chikungunya virus (CHIKV). In humans, alphaviruses can cause acute infections marked by high viremia and symptoms, including fever, rash, debilitating joint pain, encephalitis, and even morbidity [3,4]. Alphaviruses are arboviruses and are typically disseminated to humans by *Aedes aegypti* and *Aedes albopictus* mosquitos. The global spread of alphaviruses is thought to arise from a combination of expanding mosquito populations [5], adaptation of alphaviruses to new mosquito vectors [6,7,8,9], and increased international travel. Currently, there are no licensed anti-viral therapies to treat alphavirus infections, but there are promising candidate small molecule inhibitors and antibody therapies [10,11,12]. Several vaccine candidates are in clinical trial [13,14], although to date, there are no licensed alphavirus vaccines.

Alphaviruses assemble into highly organized particles that bud from the plasma membrane of infected cells. Much of our understanding comes from using the alphaviruses Sindbis (SINV) and Semliki Forest (SFV) viruses as experimental models in mammalian cell culture systems. While the results can be widely applied to the genus, some differences between virus species and cell types exist, including differences between vertebrate and invertebrate systems. Here, we will review what is known about alphavirus assembly and budding, as well as discuss recent updates on cell-to-cell transmission of alphaviruses. We will conclude this review by highlighting important unknowns in the alphavirus exit pathway. We apologize to our colleagues whose work we were not able to cite due to space limitations. Please refer to other reviews in this Special Issue “Advances in Alphavirus Research” for more information on other topics within the alphavirus life cycle.

## 2. Overview of The Alphavirus Life Cycle

**Figure 1:**
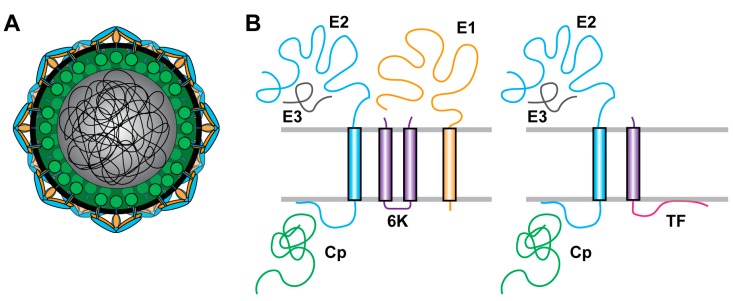
Schematic diagrams of alphavirus structural proteins. (**A**) Cartoon illustration of an alphavirus particle. E2 (blue) and E1 (orange) assemble into trimers of heterodimers embedded in the viral membrane bilayer (black). E2 directly interacts with capsid protein (green), and capsid protein assembles with the genomic RNA (enclosed dark gray sphere and lines) to form the viral nucleocapsid. Components not to scale; (**B**) cartoon illustration of the major (left) and minor (right) mature structural protein translation products. E3 is shown in grey, 6K in purple, transframe (TF) in purple and pink, and the other proteins are colored as in (**A**). Proteins not to scale.

Alphaviruses are enveloped viruses that assemble into small (~70 nm), spherical particles with T = 4 quasi-icosahedral symmetry [1]. The virion’s outer protein shell is made up of a lattice composed of 240 heterodimers of the viral envelope proteins E2 and E1 (Figure 1A). These heterodimers are organized into 80 trimers on the virion surface, giving the virus particle its “spikey” appearance. Both E2 and E1 are transmembrane glycoproteins, and E2’s C-terminal endodomain directly contacts the virus’s nucleocapsid (NC) core. The NC core is composed of 240 copies of capsid protein (Cp) arranged in an icosahedral lattice around the virus’s ~11.5 kb positive sense, single stranded RNA genome (gRNA).

Alphaviruses enter cells by binding proteinaceous receptors at the cell surface and undergoing clathrin-mediated endocytosis [15]. The specific receptor that is used varies between alphavirus species. The receptor for SINV is NRAMP2 (Natural Resistance-Associated Macrophage Protein 2) [16,17], while the receptors for other alphaviruses (SFV, CHIKV, etc.) are not yet identified. Attachment factors, such as heparan sulfate proteoglycans, can facilitate cell surface binding. After internalization, the virus-containing endocytic vesicle becomes increasingly more acidic during endosome maturation. Low pH triggers a series of conformational changes that cause E2/E1 dimer dissociation, insertion of E1’s fusion loop into the endosomal membrane, and E1 homotrimer formation, thus driving fusion between the viral and cell membranes. For a detailed review of alphavirus entry, including the work of other groups, please refer to [15,18].

Fusion between the viral and endosomal membranes deposits the virus’s NC into the cytoplasm. NC disassembly is incompletely understood, but it is facilitated by interactions with ribosomes [19,20,21,22]. The gRNA is directly translated to produce the nonstructural proteins 1, 2, 3, and 4 (nsP1, 2, 3, and 4). Through an intricate series of steps, the nsPs assemble a replication complex to produce more copies of the gRNA through a negative strand RNA intermediate. Viral RNA synthesis occurs within membrane spherules located on the plasma membrane and on cytopathic vacuole type I (CPVI) structures [23,24,25]. CPVI are membranous replication structures induced during viral infection. For a detailed review of RNA replication, please refer to [26,27].

In addition to producing more gRNA, the nsPs also produce a subgenomic RNA (sgRNA) using an internal promoter corresponding to a different open reading frame. The sgRNA corresponds to the last ~1/3 of the genome and is translated to produce a polyprotein precursor of the structural proteins. These consist of Cp, p62 (the E2 precursor, termed p62 in SFV and pE2 in SINV), 6K, E1, and transframe (TF) (Figure 1B). Cp is first to be translated and autoproteolytically cleaves itself from the nascent polyprotein [28,29,30]. Once released, Cp specifically packages the gRNA and assembles with it into NC. A series of signal sequences and transmembrane domains then mediates the translocation and topology of p62, 6K, and E1 in the ER [1,31,32]. Signal peptidase cleavage releases the individual proteins, which are posttranslationally modified as they traffic through the secretory pathway [33,34,35,36,37]. p62 and E1 form a heterodimer that protects E1 from exposure to low pH during trafficking (reviewed in [18]). Furin cleavage of p62 late in the secretory pathway produces mature E3 and E2 [38,39,40,41]. Ribosome frameshifting within the 6K coding region causes the translation of a (−1) open reading frame corresponding to the TF protein [42]. The structural proteins and the gRNA assemble into the viral particle at the plasma membrane and bud from the cell.

## 3. General Principles of Assembly and Budding

Alphavirus budding is temperature and pH dependent, with optimal budding occuring at physiological temperatures and at a neutral to mildly alkaline pH [43,44]. Budding from the cell surface occurs at an approximately constant rate and for as long as ~2 h in cell culture without the additional transport of new envelope proteins to the plasma membrane [43]. Virus assembly and budding require Cp-E2 binding, E2/E1 heterodimer formation, pH protection of E1 by p62/E3-E2, and spike lattice assembly (c.f. Section 4 for more detail). Additionally, although not being explicitly required, budding is significantly enhanced by 6K, TF, and cholesterol through unknown mechanisms [43,45,46,47,48,49,50]. Particle assembly and budding do not require p62 cleavage [39,51,52,53], nor do they require packaging of the genomic RNA [54,55,56]. However, the low abundance of p62 in particles and the high fidelity of gRNA packaging in an infected cells suggest that alphaviruses have evolved strategies to ensure that budding particles are infectious (i.e., have high specific infectivity). Recent work demonstrated that infectious microvesicles containing viral RNA and envelope proteins can form in the absence of Cp, but where quantitative analyses have been performed, this phenomenon occurs 100,000–1,000,000 times less efficiently than wild type virus particle budding [57,58], further emphasizing the efficiency of alphavirus assembly and budding under true infection conditions.

### 3.1. What Are the Minimal Requirements for Assembly/Budding?

Virus-like particles (VLPs) can be efficiently produced by the expression of the complete structural polyprotein in a mammalian or insect cell expression system [59,60,61]. Budded VLPs are structurally similar to fully infectious alphavirus particles and have comparable buoyant density [60,62]. All the critical interactions that are necessary for assembly and budding of viruses (c.f. Section 4) should also apply to VLPs, while the observed differences are intriguing.

A key difference between viruses and VLPs is the lack of a genome in VLPs, rendering them noninfectious. Rather, VLPs package cellular RNAs inside their NC cores [60,62,63]. In the absence of the non-structural proteins and the gRNA, the Cp domains that selectively bind the gRNA now have the ability to bind cellular mRNAs to help aid in their assembly (see Mendes and Kuhn review for more Cp-gRNA details). It is also important to note that Cp mutants that contain deletions or mutations in the N-terminal RNA-binding domain, including the deletion of the entire N-terminal domain of VEEV Cp, are capable of producing VLPs that are similar in structure to WT virions [64,65,66]. Interestingly, Snyder et al. observed a difference in NC assembly in infected cells vs. cells expressing the structural proteins [67]. Cp mutants that are unable to assemble NCs in infected cells nonetheless form NCs in structural protein expressing cells. Unlike infected cells, the structural protein-expressing cells do not contain the replication complex or CPVI, and thus important differences may affect the organization and/or the transport of the viral proteins in infected vs. uninfected cells.

Similar to virus budding [44], VLP yield increases at extracellular pH values >~7.0 [60]. Studies in the CHIKV VLP system have shown that particle production is promoted by mutations that stabilize the E2-E1 heterodimer [63]. Similar increases in VLP yield were produced by an antibody to E2 domain A [62], or by mutation of the p62 furin cleavage site [60]. All of these methods of VLP enhancement support the idea that stabilization of the prefusion form of the E2/E1 dimer increases the VLP yield.

### 3.2. What Drives Budding?

Cp-E2 interactions are required for budding, but there has been much discussion over what specifcally drives viral particle assembly and budding. Alphavirus particles have two layers of icosahedral symmetry: one from the spike proteins on the surface, and one from the NC core. Because abundant NCs are observed in the cytoplasm of infected cells, one model has been that pre-assembled NCs, which already have an icosahedral architecture, bind E2 at the plasma membrane to induce spike lattice formation and particle budding [55]. In support of this, microinjection of in vitro assembled core-like particles, which also show icosahedral symmetry [68], induces some particle budding, suggesting that the NC core can drive budding [69,70]. Data challenging this model come from Cp mutants that do not preassmble cytoplasmic NCs, and therefore would not be able to impose their symmetry onto the spike proteins. These Cp mutants still assemble viral NC and produce viral particles, albeit less efficiently than wild type [71,72,73]. This suggests that the envelope proteins can induce NC formation and drive particle budding. Without more precisely teasing apart their specific contributions, we can conclude that both preassembled NCs and envelope protein lateral interactions can promote particle budding. It remains unclear if host factors play a role in membrane deformation or membrane scission [74] (c.f. Section 5 for more detail).

### 3.3. Where Does Budding Occur?

Alphaviruses assemble and bud from the plasma membrane of the cell body and from virus-induced intercellular extensions [43,75,76,77,78,79] (c.f. Section 6 for more detail). Similar to mammalian cells, electron microscopy and biochemical studies demonstrate alphavirus budding from the plasma membrane of mosquito cells [43,45]. Budded virus is also observed in cytoplasmic vesicles in mosquito cells (e.g., [76]); it is not yet clear whether these structures represent true sites of internal budding or accumulations of endocytosed virus (see [80] for review). Host proteins are typically excluded from budding sites and generally are not found in viral particles; this is not surprising given that alphaviruses assemble highly symmetrical and organized particles [77,78,81,82]. Exclusion of host proteins suggests that budding may take place at specialized sites on the plasma membrane. In support of this, it has been shown that E2 accumulates in patches at the plasma membrane in a Cp-dependent manner [77], and transmission electron microscopy (TEM) studies have also observed evidence of localized budding sites at the plasma membrane [83,84,85]. Additionally, TEM and scanning electron microscopy analyses of infected cells showed copious budding viral particles at intercellular extensions and filopodia structures [75,76,77,78]. These extensions and filopodia could represent highly specialized budding locations.

## 4. Critical Interactions during Assembly and Budding

In the following section, we will describe interactions between viral structural proteins that are important for virus assembly and budding. We will discuss for each interaction pair: (1) what is known about the structural/biochemical basis of these interactions and their functional significance; and, (2) what is known about when and where these critical interactions occur in the infected cell. Please refer to Mendes and Kuhn’s review on NC assembly and gRNA packaging and Ramsey and Mukhopadhyay’s review on 6K/TF [47] within this Special Issue for detailed information on those respective critical features of alphavirus assembly.

### 4.1. Cp-E2 Interaction Is Required for Virus Assembly and Budding

#### 4.1.1. Structural Basis and Functional Significance

Cp is a ~35 kDa cytoplasmic protein that can be divided into two large sub-domains: a polybasic N-terminal region that is predominantly unstructured, and a structured C-terminal serine protease domain [29,30,86,87]. Cp’s C-terminal protease domain adopts a chymotrypsin-like fold composed of two β-barrels with a hydrophobic cleft between them [29,30]. E2 is a ~40–50 kDa transmembrane glycoprotein with a large ectodomain composed of three immunoglobulin-like domains, and a small (~30 amino acids) endodomain [88,89]. Cryo-EM reconstructions of alphavirus particles demonstrate a direct 1:1 interaction between Cp and E2, with E2’s endodomain extending into the hydrophobic cleft within Cp’s protease domain [90,91,92]. In SINV, three important contact regions were observed between Cp’s hydrophobic cleft and E2’s endodomain: region I between E2 391–395 and Cp 157–162; region II between E2 397–402 and Cp 247, 166, and 180; and, region III between E2 410–417 and Cp 249–253 [90]. Similar contacts between E2 and Cp are observed for other alphaviruses [91,92,93]. After extending into Cp’s hydrophobic cleft, E2’s endodomain loops back towards the lipid membrane in a hairpin-like fashion, with palmitoylation of conserved endodomain cysteines anchoring E2’s C-terminal tail to the membrane [37,94].

Extensive mutational and functional analyses have been performed to probe the interactions between E2 and Cp, which are essential for the budding of viral particles [55]. Synthetic peptides corresponding to the E2 endodomain bind Cp/NC in vitro and in cells, and can inhibit virus budding [95,96,97]. Mutation of conserved residues in E2’s endodomain cause assembly and budding defects [98,99,100,101]. For example, the substitution of a conserved tyrosine residue (SFV E2 Y399) with a non-hydrophobic residue causes a strong budding defect [94]. Mutations of the conserved endodomain “YAL” motif or the conserved endodomain cysteines also produced virus assembly and/or budding defects. TEM analyses show that cells infected with these mutants may display multi-cored budding viral particles, or lack NCs associated with the plasma membrane and with cytopathic vacuole type II (CPVIIs) [94,99,100,101,102]. CPVIIs are membranous structures with abundant NCs that are associated with their cytoplasmic face, and have been hypothesized to be involved in structural protein trafficking (see Section 4.1.2) [103]. In vitro binding studies using E2 endodomain peptides with purified core-like particles (CLPs), cellular NCs, and viral NCs showed decreased binding of mutant peptides when compared to WT E2 peptide [99], supporting the in vivo budding phenotypes. These studies also provided supporting evidence that electrostatic interactions, in addition to hydrophobic ones, promote binding between E2 and Cp/NC. It is worth noting that the in vitro binding of most mutant peptides to Cp/NC was not completely abrogated even though they showed striking growth phenotypes in vivo [99]. This suggests that Cp/NC binding may be more complex in vivo.

#### 4.1.2. When and Where

The intracellular location and timing of the initial Cp-E2 interaction during virus assembly and the dynamics of this interaction during the exit pathway are unclear. One important factor is the physical accessibility of E2’s endodomain for Cp binding. E2 is originally translated with two transmembrane domains, where the second transmembrane domain directs co-translational translocation of 6K into the lumen of the ER [104]. Cleavage by signal peptidase releases 6K from E2/p62, enabling E2’s C-terminus to traverse across the lipid bilayer and into the cytoplasm for Cp binding [105]. E2’s endodomain is also palmitoylated at cysteine residues, and palmitoylation is thought to orient the endodomain in a Cp-competent binding conformation, in keeping with the budding defects of E2 cysteine mutants, as discussed above [37,94]. Palmitoylation most likely occurs after exit from the ER and delivery to the Golgi compartment [106], suggesting that Cp and E2 could interact as soon as E2 is transported to the Golgi. A mutation in the SINV E2 endodomain also affects NC assembly, suggesting that Cp and E2 could interact at an early stage prior to NC formation [67].

While it is known the E2/E1 heterodimer is transported via the secretory pathway to budding sites at the plasma membrane, it is unclear how Cp/NC is targeted there. Cp/NC could passively diffuse in the cytoplasm until it contacts E2’s endodomain at the plasma membrane. Alternatively, Cp/NC could be actively transported to budding sites followed by E2 association, or transport could occur by co-transport of Cp with E2 to the plasma membrane. Virus production is significantly more efficient when the structural proteins are expressed as a polyprotein in *cis* vs. when Cp and the envelope proteins are equivalently expressed from separate subgenomic promoters [55]. One explanation for this phenomenon could be that Cp binds E2 in *cis* soon after translation, and that the complex is then co-transported to the plasma membrane via the secretory pathway. Imaging studies showed Cp and E2 co-localizing in small (0.1–0.5 µm) motile cytoplasmic puncta, supporting a possible co-transport mechanism [99,107]. Another model proposes that CPVIIs co-transport NCs and E1/E2 glycoproteins to the plasma membrane [103]. CPVIIs are membranous structures of dimensions ~100–200 nm by ~1–2 µm [103]. CPVII membranes are thought to be derived from the medial/trans Golgi [108], but to date, there are no specific markers and identification is based on morphology. The association of the NC with the outer surface of the CPVII is mediated by Cp-E2 interactions, as it is blocked by E2 endodomain mutations [94,101,105]. It is unclear whether VLP systems also produce CPVIIs. EM tomography studies showed that CPVIIs contain an internal helical tubular array of E1/E2 glycoproteins arranged similarly to the “spikes” on mature virus [103]. It is hypothesized that CPVIIs represent pre-assembled NCs and E1/E2 spikes that are co-transported to the plasma membrane. However, while CPVIIs are sometimes visualized near the plasma membrane [103], to date there is no direct evidence for their role in delivering structural proteins during budding, and more functional studies are needed.

### 4.2. E2/E1 Heterodimer and Lattice Formation Are Required for Assembly and Budding

#### 4.2.1. Structural Basis and Functional Significance

**Figure 2:**
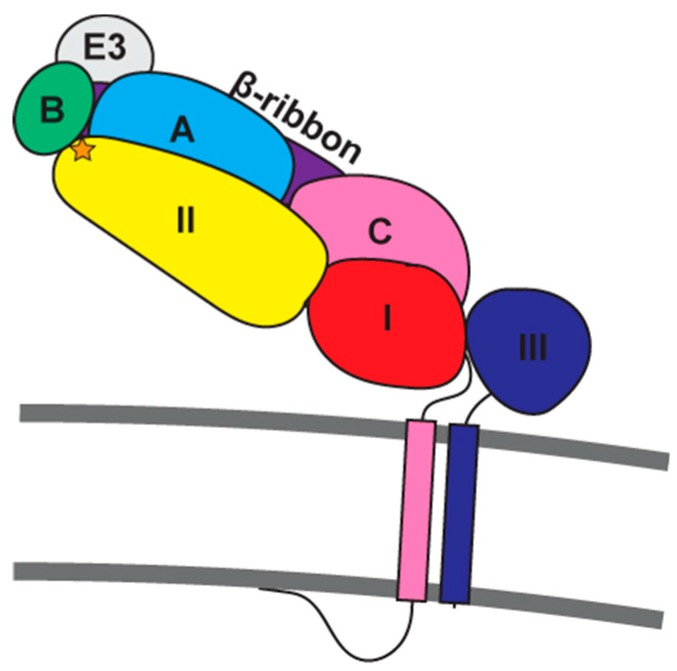
Cartoon illustration of the p62-E1 heterodimer. E3 is shown in light gray. E2 domain A is shown in cyan, domain B in green, domain C in pink, and the B-ribbon connector in purple. E2 subdomain D is not shown. E1 domain I is shown in red, domain II in yellow, and domain III in blue. E1's fusion loop is shown as an orange star. E2 and E1 transmembrane helices are shown as rectangles colored according to their respective preceding domains. The lipid bilayer is depicted as dark gray lines.

The E1 ectodomain is composed of three β-sheet-rich domains (DI-DIII), where DI links membrane-proximal DIII to distal DII, and the tip of DII contains the fusion loop [89,109] (Figure 2). The E2 ectodomain is composed of three immunoglobulin-like domains (A, B, and C), where domain C is membrane-proximal, domain A is in the center, and domain B is membrane-distal. A β-ribbon connector links domains A to B and B to C, and a recently described subdomain (D) precedes the transmembrane helix [91]. Three E2/E1 heterodimers assemble into a right-handed helix to form the spike, with E2 forming intra-spike contacts and E1 forming inter-spike contacts [110,111]. The heterodimer interactions bury about 2500 A^2^ of the protein surfaces, reflecting extensive contacts between the two proteins. Most of these contacts occur between E1 DII and E2 domain C and the β-ribbon connector, although extensive contacts also occur between E1 DII and E2 domains A and B [88,89]. Notably, the fusion loop from E1 DII extends into a groove made by E2 domains A and B, where E2 forms hydrogen bonds to the E1 fusion loop backbone [89]. This interaction is thought to be regulated by E2 histidine residues that are not conserved in primary sequence, but are clustered within the E1 fusion loop-interacting surfaces on domains B and A [89]. While most interactions between the two spike proteins occur through their ectodomains, E1 and E2 transmembrane domains also interact in a coiled coil-like fashion within the lipid bilayer [62,91,111].

For efficient virus assembly and budding to occur, correctly assembled E2-E1 heterodimers must form. Mutations that perturb heterodimer formation perturb virus budding. For example, the mutations in the E1 fusion loop can cause decreased E2-E1 heterodimer stability and produce a budding defect [112]. Additionally, mutations in the transmembrane domains affect heterodimer stability and cause budding defects [113]. E2-E1 heterodimers trimerize to further assemble into an icosahedral lattice. How this happens remains unclear, and it has been difficult to dissect lattice formation independent of heterodimer formation/stability, suggesting that they are tightly linked [114]. Although Cp also forms an icosahedral lattice in the NC structure, the envelope protein lattice can form without pre-assembled NCs [71,72,73].

#### 4.2.2. When and Where

The p62-E1 heterodimer forms *in cis* shortly after translocation into the ER, matures to the E2/E1 heterodimer after furin cleavage in a post-Golgi compartment [41], and is maintained throughout transport to the plasma membrane [115,116,117,118]. While E1 generally requires E2 for transport to the plasma membrane, E2 can transport to the plasma membrane without E1 present [119,120]. Crosslinking studies demonstrated that higher order assemblies of E2-E1 heterodimers are present in infected cells [116]. Such E2-E1 oligomers could be crosslinked soon after synthesis, presumably within the early compartments of the secretory pathway [121]. These oligomers were of a size that is consistent with hexamers, possibly reflecting an early stage of lattice assembly. However, the exact biochemical nature of these crosslinked oligomers is unclear, as well as the quaternary structure that they assemble. More work needs to be done to understand the pathway of E2-E1 lattice formation.

### 4.3. p62(E3-E2) and E1 Interactions Are Required to Prevent Premature Fusion in the Exocytic Pathway

The immature p62/E1 heterodimer is acid resistant [122] and protects E1 from prematurely fusing in the acidic environment of the trans-Golgi network (~pH 6.0). pH protection of E1 is mediated by E3, which structural studies have shown binds E2 and stabilizes the interaction between E2 domains B and A with E1’s fusion loop, thus preventing the loop’s exposure to the acidic environment [89,123]. A conserved tyrosine residue in E3 facilitates this interaction in a clade-specific manner [124] and is critical for pH protection [125]. Furin cleaves p62 after the tetrabasic motif “RHRR” late in the exocytic pathway to produce the mature E2/E1 heterodimer [39,41,126], but cleavage is not required for particle assembly/budding, and either furin cleavage site mutations that prevent processing or infection of furin-deficient cell lines still support particle production [39,51,52,53,127]. After cleavage E3 remains bound to E2 in the low pH of the secretory pathway and is released at extracellular neutral pH [128]. This E3 dissociation causes the E2/E1 heterodimer to become sensitive to the pH range of the endosome, thus priming the mature virus for fusion and infection [122,128]. Taken together, it is clear that alphaviruses have evolved elegant mechanisms to regulate protein-protein interactions in response to changes in cellular pH.

### 4.4. gRNA Packaging by Cp Is Required for Infectious Virus Assembly

#### 4.4.1. Structural Basis and Functional Significance

Cp’s protease domain is arranged into a lattice of pentamers and hexamers, called capsomers, within the icosahedral lattice. Few inter-capsomer contacts, but many intra-capsomer contacts, are made between neighboring Cp’s [62,90,91]. Cryo-EM studies visualize part of the N-terminal polybasic domain extending from the protease domain inward toward the core of the virion, where the polybasic residues are thought to interact with the negatively charged gRNA. Although significant density is observed, neither Cp’s N-terminal domain nor the gRNA have been resolved by cryo-EM, possibly reflecting heterogeneity or flexibility in the way these two components interact in the NC structure.

Cp specifically packages the gRNA to assemble into NC in the cytoplasm. gRNA packaging is favored over sgRNA and cellular RNAs, even though they are present in molar excess over the gRNA. How specificity is achieved is not well understood, but elegant studies of defective interfering particles and replicon systems of specific alphaviruses identified nucleotide sequences (packaging sequences) that promote their gRNA packaging [129,130]. Studies with VLPs and replicon systems have further shown that Cp is capable of packing other RNAs into particles in cells [54,55,56], but the high specificity for gRNA in infected cells strongly suggests that additional factors promote specific packaging. The coordination of gRNA packaging with Cp oligomerization and NC assembly is also not understood. Many studies have been performed to dissect Cp’s role in these events and have identified Cp sub-domains and residues important for packaging specificity and NC assembly [64,65,73,131]. Additionally, several in vitro studies have led to the hypothesis that Cp initially dimerizes on the PS and thus nucleates Cp oligomerization and NC assembly [132,133,134] (see also the review from Mendes and Kuhn). In some cell types, host rRNA binding proteins are also packaged into the core, and appear to enhance the translation and synthesis of the gRNA during infection [135].

#### 4.4.2. When and Where

Pulse-chase studies of NC assembly by gradient analysis showed that Cp assembles into NC within several minutes after synthesis [19,22]. Ribosomes are thought to facilitate NC assembly [19,22], but additional studies are needed to determine if they play a direct role in NC formation. It is also unclear if there is a specialized intracellular location for gRNA packaging and NC assembly. Morphological observations show that Cp and NCs are highly abundant throughout the cytoplasm and also in association with membranes (CPVIIs and the plasma membrane). Additionally, NCs can sometimes be observed near CPVI structures [76,79,136]. The functional significance of these morphological pools, such as whether they represent localized NC assembly and which are eventually incorporated into virus, is not known.

### 4.5. 6K/TF Promote Virus Budding through Unknown Mechanisms

Due to TF’s recent identification, previous phenotypes from 6K mutations will need to be carefully dissected to determine which effects are attributable to 6K, TF, or both proteins. 6K and TF are both important for virus budding and cause growth defects if deleted or mutated [42,48,49,50,137]. Both proteins can be detected in sub-stoichiometric amounts in virus [42,49,138], although recent work has shown it is TF, and not 6K, which is predominantly incorporated into virions [42,137]. Possible functions for 6K and TF include regulating Cp-E2 binding, regulating E2-E1 heterodimer formation/stability/trafficking, modulating membrane curvature, and acting as ion channels [49,98,139,140,141,142]. The precise functions of 6K and TF in virus budding are not known, and it is clear that there is still much to learn about these proteins in the alphavirus exit pathway (see also the review from Ramsey and Mukhopadhyay in this issue).

## 5. Host Factors Involved in Assembly and Budding

While much has been learned about the viral protein interactions that produce the completed infectious particle, much less is known about the role of host proteins in this process. In this section, we consider the roles of host factors in the transport of the viral components to the budding site and in promoting and inhibiting virus release.

### 5.1. Host Factors that Promote Exit

Alphaviruses use the cellular secretory machinery to transport their envelope proteins to the plasma membrane [1]. A recent siRNA screen identified several host factors in the actin-remodeling pathway that promote alphavirus glycoprotein transport to the plasma membrane [143]. Microscopy studies of VEEV and CHIKV-infected cells revealed actin rearrangements and the accumulation of actin clusters in the cytoplasm late in infection, and the colocalization of E2 with these actin foci and along actin filaments. It was proposed that E2/E1-containing CPVII vacuoles originate from the TGN with the involvement of Arf1 and Rac1, and are trafficked to the cell surface along actin filaments by a mechanism involving Rac1, Arp3, and PIP5K1-α. This mechanism could explain the trafficking of the alphavirus glycoproteins to localized sites of budding. The importance of this pathway and how the actin cytoskeleton is co-opted will be interesting areas for future research.

A number of enveloped viruses recruit the ESCRT (endosomal sorting complexes required for transport) machinery to mediate the scission of the particle from the membrane [144]. In contrast, alphavirus budding is independent of ubiquitin and VPS4 activity, and thus occurs via an ESCRT-independent pathway [74]. It is not known if there are alternative mechanisms that explain alphavirus scission, but we can speculate on several possibilities, which may not be exclusive. An as yet unidentified cellular factor could mediate the final step of scission. Alternatively, the extensive protein interactions among the envelope proteins and the NC could be sufficient to drive both membrane curvature and scission [145,146,147]. It is also possible that particle scission could be promoted by a membrane-associated viral protein that interacts with and inserts a hydrophobic domain into the cytoplasmic face of the forming virus membrane [145]. Clearly, much remains to be investigated for this membrane scission step.

### 5.2. Host Factors that Inhibit Exit

Tetherin/BST-2 is an interferon-inducible host membrane protein that restricts enveloped virus release by direct tethering of budded particles to the plasma membrane [148,149]. Even though alphaviruses are highly organized and exclude bulk host membrane proteins from budding sites, tetherin inhibits alphavirus release [150,151]. Tetherin expression in mice that are infected with CHIKV was found to be protective in lymphoid tissues, and also acted to restrict spread to distant tissues and regulate the inflammatory response [152]. SFV release was more efficiently inhibited by the long isoform of tetherin vs. the short, which lacks 12 residues in the N-terminal cytoplasmic tail [151]. Since tetherin has broad antiviral activity, many viruses have evolved countermeasures against it by encoding viral antagonists [148,149]. It has been suggested that nsP1 downregulates tetherin expression to promote VLP release [150]. More direct assays of particle release and rigorous studies of the tetherin isoform requirement will help to define the mechanism of tetherin inhibition and possible antagonism by alphavirus proteins.

## 6. Cell-to-Cell Transmission

Much of what virologists have learned about virus entry and exit is based on studies of the biology of cell-free virus. Alphaviruses are a classic example of viruses that produce highly infectious cell-free virus particles. However, initial evidence for cell-to-cell CHIKV transmission was presented as early as 1970 [153]. CHIKV infections in humans are usually associated with brief viremia and then rapid viral clearance within one to two weeks due to the robust humoral response, which would act as a barrier to cell-free virus infection. In spite of this, reports have described the persistence of CHIKV-specific IgM responses in humans [154] and long-term detection of CHIKV antigens in macrophages of non-human primates [155], suggesting that CHIKV might establish chronic infection capable of evading immune clearance. Viruses can escape antibody responses by evolving mutations that abolish antibody neutralization or by utilizing cell-to-cell spread as an alternative, faster evasion strategy. The first description of cell-associated CHIKV dissemination as a mechanism of antibody escape was in 1970 by Hahon and Zimmerman [153]. They observed the presence of foci containing several infected cells in the presence of potent antiviral serum that completely blocked infection by cell-free virus, suggesting cell-to-cell virus transmission. In the absence of antiviral serum, they observed a mixture of foci and individually infected cells in certain cell lines, but not others, and concluded that the mode of virus transmission may be dependent on the cell line and host factors involved.

**Figure 3:**
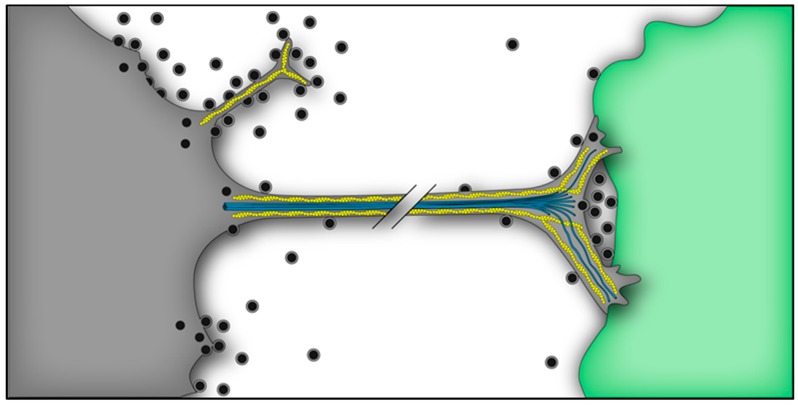
Schematic model of specialized budding sites of an alphavirus infected cell (gray). Alphaviruses assemble and bud from localized patches at the plasma membrane of the cell body, where host proteins are excluded and Cp/NC serves as a scaffold to accumulate E2/E1 glycoproteins. Dramatic cytoskeletal remodeling occurs during infection, and short filopodia-like extensions (top) and long intercellular extensions (middle) are observed. Short extensions contain F-actin (yellow) only, while intercellular extensions contain F-actin and tubulin (blue) structures. Nascent virus particles bud along the short extension, which may facilitate dissemination from the infected cell. An intercellular extension projects from the infected cell and makes preferential contact with an uninfected cell (green). The contact site forms a stable, flattened tip on the target cell plasma membrane (right), producing a protected pocket where nascent particles bud and are subsequently internalized by the uninfected cell. Thus, intercellular extensions are close-ended membrane bridges that mediate cell-to-cell transmission. Host determinants involved in modulating the cytoskeleton, forming/stabilizing extensions, and promoting cell-to-cell virus transmission are currently unknown.

Alphavirus infection induces a dramatic cytoskeletal remodeling of the host cell, including in particular the formation of two different types of filopodia-like extensions here referred to as short extensions and long intercellular extensions (Figure 3). These structures can be distinguished from one another by their length, contacts, and components. In this section, we will discuss what is known about cell-to-cell transmission of alphaviruses and the role of these extensions in facilitating this route of transmission.

### 6.1. Short Extensions

Alphavirus-induced short extensions (Figure 3) were first described by Birdwell et al., who observed by correlative surface replica and thin-section electron microscopy that budding often occurred in filopodia-like structures extending from the cell body [84] and that virus glycoproteins were densely packed in these extensions [156]. These short extensions are ~2–7 μm in length [77,84], may contain branching at distal ends [84,157], and vary from one to several virion diameters in width [84]. They do not contain tubulin, but mostly contain F-actin, especially at the root [77,157]. nsP1 expression alone induces short extensions that appear morphologically similar to those that are induced during alphavirus infection [157]. nsP1 is present along the length of the filopodia and co-localizes with CD44 and ezrin. Membrane association as mediated by an amphipathic helix in nsP1 [158,159] or by nsP1 palmitoylation [159,160,161,162,163] can promote nsP1 localization to and induction of filopodia in mammalian cells. Some short extensions in infected cells are nsP1-postitive but E2-negative [76]. Those that are E2-positive contain all of the viral structural proteins [77], and are usually nsP1-positive, but are negative for replication complexes, dsRNA, nsP2, nsP3, and nsP4 [76]. During infection the E2 protein in short extensions becomes immobile due to its Cp interaction, plasma membrane marker proteins are excluded, and abundant virus-sized particles are observed all throughout the length of the extension [77,84,85,156]. Single particle tracking and live cell imaging showed nascent SINV particles budding from or in close association with these short extensions [75].

Cells expressing only the viral structural proteins produce VLPs but do not form short extensions [78], suggesting that the short extensions observed in virus-infected cells are promoted by nsP1. What might be the purpose of these short filopodia-like extensions? By localizing budding away from the bulk plasma membrane, they may help to avoid inhibition that is caused by the traffic of plasma membrane-associated replication complexes [164], or to prevent superinfection caused by rebinding of nascent virus back to the infected cell [75]. Thus, short extensions can be considered specialized sites of budding and production of cell-free viruses [75,77,84,85,156].

Based on all of the studies to date, it is unclear if the short nsP1-positive extensions can mediate cell-to-cell transmission or confer protection from antibody neutralization, a hallmark of such transmission. Interestingly, an SFV mutant defective in nsP1 palmitoylation produces very small plaques in HeLa cells as compared to WT virus, despite its ability to grow to high titers in liquid cultures [160]. This suggests that nsP1 localization within the short extensions could be involved in enhancing alphavirus cell-to-cell spread. Further studies are required to determine if this mutant is affected in the formation of long intercellular extensions. Mice infected with this mutant survive and show no infection of brain tissue in contrast to WT virus [160]. Thus, nsP1 palmitoylation and its localization within short extensions could be a contributing factor to SFV pathogenesis.

### 6.2. Virus-Induced Long Intercellular Extensions

Long intercellular extensions emanate from infected cells to physically contact neighboring cells [77,78] (Figure 3). These extensions are >10 μm in length, typically within the 10–60 μm range. They are tubulin and F-actin positive, but often display short branches that are tubulin and F-actin negative. They are relatively large in diameter, and are typically detected once virus structural protein production is well underway. Long intercellular extensions do not extensively contact the substratum, but when they do, they often show a flattened point of contact, and their contact with the neighboring cell also displays a flattened tip. E2, E1, and Cp staining can be detected throughout the length of the extension, without detectable exclusion of host plasma membrane proteins. They are induced by several alphaviruses (SINV, SFV, CHIKV, VEEV), including clinical isolates, and are observed upon infection of many different cell types, including mammalian fibroblasts (baby hamster kidney (BHK), 3T3) and epithelial cells (Vero, U2-osteosarcoma (U2-OS), HeLa, Chinese hamster ovary (CHO)), primary cell lines (human umbilical vein endothelial cells (HUVEC)), and mosquito cells (C6/36) [76,77,78]. There are subtle differences in the number of long intercellular extensions induced amongst alphaviruses and cell types. For example, long intercellular extensions are not observed in SFV-infected U2-OS cells or in SFV- or CHIKV-infected CHO cells, while SINV induces extensions in both of these cell types. While the reasons for these differences are currently unknown, we speculate that they could reflect the presence or absence of specific host proteins necessary for the formation or stabilization of the contacts of the infected and target cells.

Microscopy studies show that the intercellular extensions do not transfer a soluble cytosolic dye (5-chloromethylfluorescein diacetate, ~465 Da) or a freely diffusing plasma membrane marker between an infected and target cell [78]. These data indicate that the intercellular extensions do not fuse with the target cell and do not mediate membrane or cytoplasm continuity between the two cells. In the case of retroviral cell-to-cell transmission, long filopodia from non-infected cells form contacts with infected cells through the interaction of the viral envelope protein on the infected cell with the virus receptor on the target cell [165]. A process termed frustrated phagocytosis mediates the stable interaction and internalization of the filopodia tip via endocytic factors, while released virus then “surfs” along the filopodia by retrograde actin transport from the infected cell to the target cell [166]. In contrast, alphaviral intercellular extensions originate exclusively from infected cells and form stable physical contacts with neighboring cells [78] (Figure 3). In the case of SINV, the virus receptor NRAMP2 and attachment factor heparan sulfate are not involved in the formation or stabilization of intercellular extensions, and are not required for cell-to-cell transmission [78]. Stabilization of intercellular extensions does not appear to utilize frustrated phagocytosis, since no accumulation of endocytic markers is observed at the contact sites. Presumably, other host factors on the infected and target cell promote the attachment and further stabilization of these intercellular extensions, but these remain to be identified.

Studies with SINV and SFV show that the induction of intercellular extensions requires the delivery of E2 to the cell surface and its interaction with the Cp [78]. Cells that are infected with virus mutants that have defects in formation of cytoplasmic NCs or that perturb the stability of the E2-E1 heterodimer still induce the formation of long extensions similar to WT, as long as they support E2-Cp interaction at the plasma membrane [77,114]. In contrast, cells infected with the non-budding mutant SINV E2 Y400K, which is unable to form a stable E2-Cp interaction, do not form intercellular extensions [77,78]. Studies with a temperature sensitive budding mutant that maintains the interaction of E2 with Cp at the cell surface show that budding per se is not required for extension formation. In the absence of virus infection, the expression of the viral structural proteins efficiently induces long intercellular extensions, with a similar requirement for cell surface E2-Cp interaction [78]. Co-culture experiments demonstrate that the extensions induced by the viral structural proteins are preferentially targeted to and/or stabilized with non-expressing cells.

Live-cell imaging of co-culture experiments suggests several possible mechanisms for the formation of intercellular extensions. Intercellular extensions were observed to initiate de novo from a virus-infected cell and make contact with a neighboring uninfected cell [77]. Intercellular extensions were also observed to form after an infected cell and an uninfected cell make initial cell contact and then subsequently move apart from one another [78]. In each case, the intercellular extensions remain quite stable, even in the face of continued movement of both cells in the pair. At this point, it is unclear if there is a preferred mechanism and whether the intercellular extensions initiate as filopodia or retraction fibers, which appear very similar but have differing properties [167,168]. Interestingly, a loss of stress fibers can be observed in infected cells at late times of infection and correlates with the appearance of filopodia-like extensions [164]. More studies on how the cytoskeleton is remodeled during the formation of intercellular extensions and how the viral components or particles are trafficked to the tips of the extensions will be necessary to elucidate the mechanism of extension formation.

Cell-to-cell transmission is mediated by the long intercellular extensions (Figure 3), and it is relatively insensitive to receptor down-regulation or neutralizing antibodies [78]. This cell-to-cell transmission would be promoted by preferential targeting of the extensions from an infected cell to an uninfected target cell, similar to what was observed in the VLP studies described above. We hypothesize that transmission occurs because the flattened membrane tips at the end of intercellular extensions create a protected interface between the infected and target cell. Virus budding into this space would be inhibited from diffusion into the extracellular milieu, thus generating a relatively high virus concentration and promoting virus internalization and subsequent low pH-triggered virus fusion and infection of the target cell. Such protected intercellular structures would also account for the relative resistance of the virus to neutralizing antibodies.

### 6.3. Other Possible Modes of Cell-to-cell Transmission

A cell-to-cell transmission model was proposed based on the characterization of a CHIKV E2 R82G mutant, selected for resistance to a neutralizing antibody [169]. Under conditions that block cell-free CHIKV infection, producer cells infected with the CHIKV E2 R82G mutant more efficiently transmitted infection than WT CHIKV-infected cells. Mice infected with the R82G mutant died a day earlier and contained higher viral loads in both liver and serum when compared to WT. In the presence of neutralizing antibodies, tight associations between the cell bodies of infected and target cells were observed. It was proposed that cell-to cell transmission might involve structures, termed virological synapses, mediated by cell-cell contacts, such as tight junctions. Further studies will be important to determine if such “synapses” are indeed functionally involved in alphavirus cell-cell transmission.

Mosquito cells in culture become persistently infected, and live cell imaging of such cells reveals that their plasma membranes are relatively devoid of E2 glycoprotein, while filopodia-like extensions are observed [76]. It will be interesting to further characterize these extensions and their possible involvement in establishing or maintaining persistent infection.

### 6.4. Concluding Remarks

While much remains to be determined, certain general principles of alphavirus cell-to-cell transmission via long intercellular extensions are clear. Transmission requires the budding and release of fusion-active virus particles [78]. Thus, transmission is not mediated by transfer of viral RNA, replication complexes, or CPVIIs. This is in keeping with the observed lack of cytoplasmic or membrane continuity between the infected and target cells. The SINV receptor NRAMP2 and the attachment factor heparan sulfate are not required for SINV transmission. Intercellular transmission protects the virus from neutralizing antibodies, suggesting that the nascent virus particles are somehow shielded from the extracellular milieu. The data also strongly suggest that target cell infection takes place via endocytosis and low pH-triggered fusion.

## 7. Outstanding Questions in Alphavirus Assembly and Budding

Despite years of research and great advances in knowledge, there are still many outstanding questions regarding the alphavirus exit pathway. While many of the viral requirements and critical interactions for virus assembly and budding have been defined, many questions still remain on their temporal and spatial dynamics. This includes when and where Cp and E2 interact, how they are transported, and when and where spike lattice assembly occurs. There are still major gaps in our understanding of the alphavirus exit pathway in part due to relatively recent findings, including the identification of cell-to-cell transmission of alphavirus particles via intercellular extensions. This transmission strategy has generated many questions regarding the molecular and cellular mechanisms involved, as well as the importance of cell-to-cell spread for in vivo pathogenesis, persistence, and immune evasion. Developing new imaging approaches and identifying small molecule inhibitors and antibodies that can specifically block the formation or stabilization of intercellular extensions could prove to be useful experimental tools. There are also broader, more open-ended questions on alphavirus exit, such as the identity and function of host factors that are involved in the assembly and budding of viral particles. Finally, an overarching and exciting question for the field is how addressing these fundamental gaps in knowledge can be linked to the development of new therapeutic strategies.
